# RACIAL AND ETHNIC VARIATIONS IN DEMENTIA DIAGNOSIS, SURVIVAL, AND END-OF-LIFE CARE QUALITY

**DOI:** 10.1093/geroni/igac059.1396

**Published:** 2022-12-20

**Authors:** Olga Jarrín, Zahra Rahemi, Michael Gusmano

## Abstract

In the United States most adults have a preference to die at home and is an indicator of good end-of-life care. In the context of dementia, family members and caregivers are decision makers and part of good and equitable care involves understanding cultural variation in attitudes and social norms related to dementia, death and dying, and the meaning of a good death. This symposium explores racial and ethnic variation in lifetime dementia diagnosis and end-of-life care quality indicators. The first presentation examines racial, ethnic, and geographic variation in the rarely discussed lifetime prevalence of dementia and survival time from dementia diagnosis to death using national Medicare data. The second presentation describes the relationship between end-of-life care planning and satisfaction with end-of-life care using data from the Health and Retirement Study. The third presentation describes variation in place of death, a key indicator of end-of-life care quality, by dementia diagnosis and race/ethnicity using national Medicare data. The fourth presentation examines variation in hospice use, another indicator of end-of-life-care quality, and place of death by dementia diagnosis, race, and ethnicity using national Medicare data. The symposium concludes with a presentation examining the relationship between place of death and satisfaction with care received using data from the Health and Retirement Study. The Institute for Healthcare Improvement’s Triple Aim (improving the experience of care, improving the health of populations, and reducing per capita costs of health care) serves as a lens for discussing policy and practice implications of the major findings from each presentation.

